# Hemangioendothelioma arising from the spleen: A case report and literature review

**DOI:** 10.3892/ol.2014.2693

**Published:** 2014-11-10

**Authors:** ZHENDAN WANG, LIANG ZHANG, BO ZHANG, DIANBIN MU, KAI CUI, SHENG LI

**Affiliations:** 1 Department of Hepatology, Shandong Academy of Medical Sciences, University of Jinan, Jinan, Shandong 250022, P.R. China; 2 Department of Hepatobiliary Surgery, The Third People’s Hospital of Jiaozhou, Jiaozhou, Shandong 266300, P.R. China; 3 Department of Hepatobiliary Surgery, Shandong Cancer Hospital, Jinan, Shandong 250117, P.R. China; 4 Department of Pathology, Shandong Cancer Hospital, Jinan, Shandong 250117, P.R. China

**Keywords:** hemangioendotheliomas, spleen, partial splenectomy

## Abstract

Primary hemangioendotheliomas (HEs) of the spleen are rare, low-grade borderline-malignant vascular tumors. To date, only a few splenic HE cases have been reported in adults. In infants, one 9-year-old male patient has previously been reported, and the patient succumbed to the disease shortly following surgery. Currently, the clinical treatment and prognosis of the disease remains challenging to define, due to the extremely low number of cases reported. The current report presents the case of a 9-year-old pediatric patient with splenic HE, who survived with no recurrence or complications following a partial splenectomy. Additionally, a literature review was conducted to analyze the treatment and prognosis of the disease.

## Introduction

The term ‘hemangioendothelioma’ (HE) has been used to describe a heterogenous group of vascular neoplasms, which are intermediate between benign and malignant tumors (1). Cases of HE often progress to rare metastasis and infrequent mortality, primarily arising in the soft tissues of the extremities and organs, including anterior mediastina and liver in adults (2). HEs are primarily surgically excised. HEs from soft tissues often exhibit a good prognosis, while HEs from internal organs often exhibit a relatively poor prognosis, as patients often succumb during surgery due to complications due to progress of the disease itself (3).

As an important peripheral immune organ, the spleen is rarely reported as the origin of HE tumors. To the best of our knowledge, few cases of HE have been reported to have arisen from the spleen in adults, and only one pediatric patient with splenic HE has been described (4). The current report presents the second pediatric patient to be diagnosed with splenic HE. Furthermore a literature review is conducted to summarize the clinical treatment and outcomes of splenic HE.

## Case report

**Figure 1:**
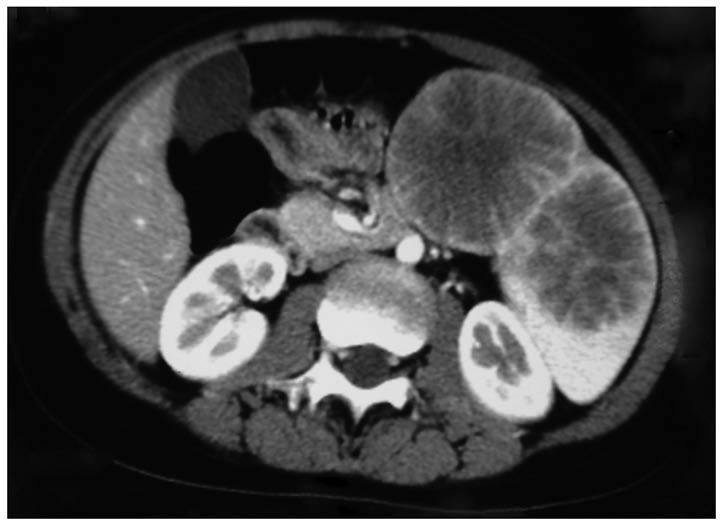
Computed tomography scan revealing two circular low density areas with clear boundaries. Circular, radiative, enhanced signals were observed at the margin of the tumor mass.

A 9-year-old female patient presented to the Department of Hepatobiliary Surgery, Shandong Cancer Hospital (Jinan, China) due to 20-day intermittent abdominal pain. No abnormalities were observed during the physical examination with the exception of an enlarged spleen (degree II) (5). Laboratory tests were normal with the exception of a slight increase in the serum levels of cancer antigen 125 (45.4 U/ml, normal range ≤35 U/ml) and white blood cells (11.5×10^9^ per liter, normal range 4–10×10^9^ per liter). Computed tomography (CT) scanning revealed two tumor masses in the lower lobe of the splenic parenchyma, with the larger one measuring 5.6 × 5.9 × 6.3 cm (Fig. 1). The images are characterized by a round, or round-like low density area with clear boundary to the normal tissues, as revealed by the plain scanning, and circular, radiative enhancement at the margin.

**Figure 2:**
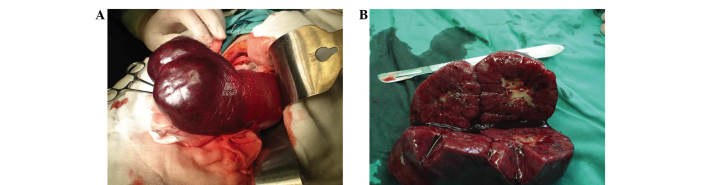
Primary hemangioendotheliomas of the spleen. (A) Two tumor masses, growing exogenously, were observed at the lower spleen pole. (B) The sizes of the tumor masses were 6.0 × 6.0 × 5.5 cm and 5.0 × 5.0 × 4.0 cm. The texture of the tumor mass was soft with a gray color in the center of the incisal surface.

**Figure 3:**
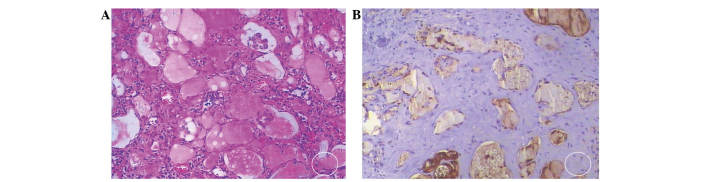
Stained sections of the tumors. (A) Hematoxylin and eosin staining indicated dispersed hyperplasia of vascular endothelial cells invading into the lumen (magnification, ×100). (B) Immunohistochemical analysis revealed that the tumor cells were positive for FVIII (magnification, 200x).

Initially a laparotomy was performed, and a rapid diagnosis of the carcinoma was conducted, which revealed borderline carcinoma with slight cellular heteromorphism. Due to this, a partial splenectomy was performed. Written informed consent was obtained from the patient’s parents. Following the surgery, a mass was cut from the lower spleen lobe. The excised section showed a well-circumscribed mass that was separate from the surrounding splenic parenchyma. Upon further observation, two tumor masses, growing in an exogenous pattern, were observed at the lower spleen pole, measuring 6.0 × 6.0 × 5.5 cm and 5.0 × 5.0 × 4.0 cm (Fig. 2). Pathological examination indicated that the tumor masses primarily consisted of capillaries and well-differentiated great vessels (Fig. 3). In addition, a large volume of liquid was contained within the great vessels, together with dispersed hyperplasia of the vascular endothelial cells (VECs), which invaded the lumen. In the immunohistochemical analysis, the tumor cells were positive for CD31+, CD34+, FVIII+, Ki-67+<1%, pan CK+ and TG−. To prevent the development of malignant carcinoma, the patient attended a check-up every three months. To date, no recurrence or deterioration has been reported in the 15 months following the surgery.

## Discussion

The diagnosis of splenic HE remains challenging as no specific clinical manifestations are evident in the early stages of development (6). With the progressive growth of the tumor, the patient may experience left upper abdominal pain, splenic swelling and an abdominal mass. In severe cases, a series of symptoms may be induced, including gastric distention, nausea, vomiting, dyspnea, shoulder pain and constipation (7–9). CT imaging is an important technique used in the diagnosis of splenic HE, which is characterized by early-stage radiative enhancement at the margin and by delayed enhancement filling the center. However, the final diagnosis of splenic HE relies on pathological examination.

To the best of our knowledge, no studies have been published that summarize the treatment efficiency of splenic HE cases following the initial report (9). In the current study, a literature review of splenic HE was conducted following a search of PubMed using the key words ‘hemangioendothelioma’ and ‘spleen’ or ‘splenic hemangioendothelioma’. A total of six cases of splenic HE were reported prior to February 2014 (Table I).

The therapeutic strategies for splenic HE have been limited as cases are rare. In adults, the most effective therapy is considered to be complete splenectomy (7–11). However, this incurs increased postoperative risks, including the possibility of post-splenectomy infection, which may occur if an infant underwent a full splenectomy. Furthermore, as the immune system of infants is not fully developed, as it is in adults, a complete splenectomy may exert negative effects on the immune system. In the current report, the tumor mass was distanced from the splenic hilum, and no adhesion to the surrounding tissues was observed, therefore, a partial splenectomy was performed.

To our best knowledge, only one case of pediatric splenic HE had been reported prior to the current case report (4). The patient, a 9-year-old male with HE of the liver and spleen, received a partial splenectomy, however, he succumbed to consumptive coagulopathy. The current case report presents the case of a 9-year-old female patient with splenic HE, who underwent a partial splenectomy. The patient showed no recurrence or complications during the 15-month follow up. This report, together with the literature review of previous cases, provides clinical guideline for clinicians with regard to the management of splenic HE. The limitation of this case report is that the exact type of HE was not determined. However, the effect of this on the treatment and prognosis of the patient is minimal.

## Figures and Tables

**Table I:** Clinical features of six cases of splenic hemangioendothelioma.

Case	Gender/age, years	Symptoms	Treatment	Follow-up	Reference
1	M/45	Enlarged spleen and brain metastasis	Resection of tumor mass	Five weeks post-onset, succumbed to the disease	4
2	M/3	Well-circumscribed mass in splenic parenchyma	Partial splenectomy	Five years, survived	5
3	M/9	HE of liver and spleen	Splenectomy and liver biopsy	Two days, mortality due to consumptive coagulopathy	3
4	F/36	Kaposiform HE	Splenectomy	Six months, no recurrence or metastasis	6
5	F/67	Composite HE arising from the spleen	Splenectomy and chemotherapy	Not available	7
6	F/28	Splenic littoral cell HE, hepatic metastasis	Splenectomy	Not available	8

M, male; F, female.
